# Supplementary material for: Identification of Cassiopea sp. in Lake Macquarie, Australia and revision of the taxonomic status of Cassiopea maremetens Gershwin, Zeidler & Davie, 2010 (Cnidaria: Scyphozoa: Cassiopeidae)
Source: PeerJ. 2025 Jul 18;13:e19669. doi: 10.7717/peerj.19669 (PMC12278942; doi:10.7717/peerj.19669)
Supplement: Supplemental Information 1 — Extracted from material made available by SAM, AM, ReefHQ or QM, or obtained from GenBank. [file peerj-13-19669-s001.docx]

| **Institution registration number** | **Species** | Locality | **Name in Tree** | **Genbank** | **Source** |
| --- | --- | --- | --- | --- | --- |
| AM G.18610 | *C. maremetens* | Lake Petite, Lake Macquarie,NSW | Lake Macquarie NSW 1 | PV365315 | This paper |
| AM G.18611 | *C. maremetens* | Lake Petite, Lake Macquarie,NSW | Lake Macquarie NSW 2 | PV365316 | This paper |
| SAM H1840 | *C. andromeda* | Angas Inlet, South Australia | *C andromeda* Angas Inlet SA 1 | PV365289 | This paper |
| SAM H3558 | *C. andromeda* | Angas Inlet, South Australia | *C andromeda* Angas Inlet SA 2 | PV365290 | This paper |
| SAM H3569 | *C. andromeda* | Angas Inlet, South Australia | *C andromeda* Angas Inlet SA 3 | PV365291 | This paper |
| SAM H3571 | *C. andromeda* | Angas Inlet, South Australia | *C andromeda* Angas Inlet SA 4 | PV365292 | This paper |
| SAM H3577 | *C. andromeda* | Angas Inlet, South Australia | *C andromeda* Angas Inlet SA 5 | PV365293 | This paper |
| SAM H3579 | *C. andromeda* | Angas Inlet, South Australia | *C andromeda* Angas Inlet SA 6 | PV365294 | This paper |
| SAM H3581 | *C. andromeda* | Angas Inlet, South Australia | *C andromeda* Angas Inlet SA 7 | PV365295 | This paper |
| AM G.18349 | *C. maremetens* | Wallis Lake,NSW | Wallis Lake NSW 1 | PV365302 | This paper |
| AM G.18344 | *C. maremetens* | Lizard Island | *C* sp Lizard Is QLD | PV365313 | This paper |
| AM G.18700 | *C. maremetens* | Gold Coast, QLD | Moreton Bay 1 | PV365298 | This paper |
| AM G.18701 | *C. maremetens* | Gold Coast, QLD | Moreton Bay 2 | PV365299 | This paper |
| AM G.18711 | *C. maremetens* | Mannering Bay, Lake Macquarie,NSW | Lake Macquarie NSW 3 | PV365317 | This paper |
| AM G.18712 | *C. maremetens* | Mannering Bay, Lake Macquarie,NSW | Lake Macquarie NSW 4 | PV365318 | This paper |
| AM G.18722 | *C. maremetens* | Karignan Creek, Lake Macquarie,NSW | Lake Macquarie NSW 5 | PV365319 | This paper |
| AM G.18725 | *C. maremetens* | Kilaben Creek, Lake Macquarie,NSW | Lake Macquarie NSW 6 | PV365320 | This paper |
| AM G.18726 | *C. maremetens* | Kilaben Creek, Lake Macquarie,NSW | Lake Macquarie NSW 7 | PV365321 | This paper |
|  | *C. maremetens* | Myuna Bay, Lake Macquarie,NSW | Lake Macquarie NSW 8 | PV365322 | This paper – arm collected from specimen in the field |
|  | *C. maremetens* | Myuna Bay, Lake Macquarie,NSW | Lake Macquarie NSW 9 | PV365323 | This paper – arm collected from specimen in the field |
| QM G.339123 | *C. maremetens* | Gold Coast, QLD | Gold Coast QLD 1 | PV365296 | This paper |
| QM G.339128 | *C. maremetens* | Gold Coast, QLD | Gold Coast QLD 2 | PV365297 | This paper |
| AM G.18075 | *C. ndrosia* | Lake Illawarra, NSW | *C ndrosia* Lake Illawarra | PV365312 | This paper |
| AM G.18143 | *C. maremetens* | Wallis Lake, NSW | Wallis Lake NSW 2 | PV365304 | This paper |
| AM G.18144 | *C. maremetens* | Wallis Lake, NSW | Wallis Lake NSW 3 | PV365305 | This paper |
| AM G.18145 | *C. maremetens* | Wallis Lake, NSW | Wallis Lake NSW 4 | PV365306 | This paper |
| AM G.18146 | *C. maremetens* | Wallis Lake, NSW | Wallis Lake NSW 5 | PV365307 | This paper |
| AM G.18147 | *C. maremetens* | Wallis Lake, NSW | Wallis Lake NSW 6 | PV365308 | This paper |
| AM G.18148 | *C. maremetens* | Wallis Lake, NSW | Wallis Lake NSW 7 | PV365309 | This paper |
| AM G.18149 | *C. maremetens* | Wallis Lake, NSW | Wallis Lake NSW 8 | PV365310 | This paper |
| AM G.18151 | *C. maremetens* | Wallis Lake, NSW | Wallis Lake NSW 9 | PV365311 | This paper |
|  | *C. maremetens* | Moreton Bay, QLD | Moreton Bay QLD 3 |  | XH00446 |
|  | *C. maremetens* | Moreton Bay, QLD | Moreton Bay QLD 4 |  | XH00447 |
| AM G.18182 | *C. maremetens* | Wallis Lake, NSW | Wallis Lake NSW 10 | PV365303 | This paper |
| AM G.20068 | *C. maremetens* | Pelican Waters, QLD | Pelican Waters QLD 1 | PV365300 | This paper |
| AM G.20069 | *C. maremetens* | Pelican Waters, QLD | Pelican Waters QLD 2 | PV365301 | This paper |
| AM G.20059 | C sp3 | Coombabah Creek, QLD | *C* sp3 Coombabah Creek 1 | PV365314 | This paper |
| AM G.20076 | C sp | Royal National Park, NSW | *C* sp Port HackingNSW 1 | PV365324 | This paper |
| AM G.20076 | C sp | Royal National Park, NSW | *C* sp Port Hacking NSW 2 | PV365325 | This paper |
|  | C sp 1 | Port Douglas, QLD | *C* sp1 Port Douglas QLD | AY319471.1 | Holland et al (2010) Global phylogeography of Cassiopea (Scyphozoa: Rhizostomeae) molecular evidence for cryptic species and multiple invasions of the Hawaiian Islands |
|  | C sp3 | Lake Alexander, NT | *C* sp Lake Alexander NT 1 | MF742136.1 | Abboud et al (2018) Global estimate of genetic and geographic differention in macromedusae implications for identifying the causes of jellyfish blooms |
|  | C sp3 | Lake Alexander, NT | *C* sp Lake Alexander NT 2 | MF742137.1 |  |
|  | C sp3 | Lake Alexander, NT | *C* sp Lake Alexander NT 3 | MF742138.1 |  |
|  | C sp3 | Coombabah Creek, QLD | *C* sp3 Coombabah Creek QLD 2 | MF742133.1 |  |
|  | C sp3 | Coombabah Creek, QLD | *C* sp3 Coombabah Creek QLD 3 | MF742134.1 |  |
|  | C sp3 | Coombabah Creek, QLD | *C* sp3 Coombabah Creek QLD 4 | MF742135.1 |  |
|  | *Catostylus mosaicus* | Port Albert, Victoria, Australia | *Catostylus mosaicus* | AY737222.1 | Dawson (2005) Incipient speciation of Catostylus mosaicus (Scyphozoa, Rhizostomeae, Catostylidae), comparative phylogeography, and biogeography in southeast Australia |
|  | *Aurelia aurita* | Boston Harbour,Massachusetts, USA | *Aurelia aurita* | AY903093.1 | Dawson (2005) Coupled biophysical global ocean model and molecular genetic analyses identify multiple introductions of cryptogenic species |
