# Supplementary material for: Identification of Cassiopea sp. in Lake Macquarie, Australia and revision of the taxonomic status of Cassiopea maremetens Gershwin, Zeidler & Davie, 2010 (Cnidaria: Scyphozoa: Cassiopeidae)
Source: PeerJ. 2025 Jul 18;13:e19669. doi: 10.7717/peerj.19669 (PMC12278942; doi:10.7717/peerj.19669)
Supplement: Supplemental Information 2 — Sequences sourced both from this study and GenBank. [file peerj-13-19669-s002.docx]

| **Institution registration number** | **Identification from source** | **Updated identification** | **Locality** | **Genbank** | **Name In Tree** | **Reference** |
| --- | --- | --- | --- | --- | --- | --- |
| AM G.18610 | *C. maremetens* | *C. xamachana* | Lake Petite, Lake Macquarie,New South Wales, Australia | PV365315 | Lake Macquarie, Australia | This paper |
| AM G.18075 | *C. ndrosia* | *Cassiopea* sp3 | Lake Illawarra, New South Wales, Australia | PV365312 | *C. ndrosia* Lake Illawarra, Australia | This paper |
| AM G.18344 |  | *Cassiopea* sp2 | Lizard Island, Queensland, Australia | PV365313 | *C.* sp.Lizard Island, Australia | This paper |
| SAM H1840 | *C. andromeda* | *C. andromeda* | Angas Inlet, South Australia, Australia | PV365289 | *C. andromeda* South Australia | This paper |
| Not registered | *C. ornata* | *C. ornata* | Singapore |  | *C. ornata* Singapore | This paper |
| Not registered | *C. andromeda* | *C. andromeda* | Israel |  | *C. andromeda* Israel | This paper |
| AM G.18349 | *C. maremetens* | *C. xamachana* | Wallis Lake, New South Wales, Australia | PV365302 | Wallis Lake, Australia | This paper |
| AM G.20068 | *C. maremetens* | *C. xamachana* | Pelican Waters, Queensland, Australia | PV365300 | Pelican Waters, Australia | This paper |
| AM G.20059 | *Cassiopea* sp3 | *Cassiopea* sp3 | Coombabah Creek, Queensland, Australia | PV365314 | *C.* sp3 Coombabah Creek, Australia 1 | This paper |
| AM G.20076 | *Cassipea sp* | *Cassiopea* sp3 | Royal National Park, New South Wales, Australia | PV365324 | *C* sp, Port Hacking, Australia |  |
|  | *Cassiopea* sp3 | *Cassiopea* sp3 | Kahuku fish pond, windward O’ahu | AY319452.1 | *C.* sp 3 Oahu, Hawaii 1 | Holland et al (2010) Global phylogeography of Cassiopea (Scyphozoa: Rhizostomeae): molecular evidence for cryptic species and multiple invasions of the Hawaiian Islands |
|  | *Cassiopea* sp3 | *Cassiopea* sp3 | Wedding Chapel, windward O’ahu | AY331594.1 | *C.* sp 3 Oahu, Hawaii 2 |  |
|  | *Cassiopea* sp3 | *Cassiopea* sp3 | Mid Pacific Golf Course pond, windward O’ahu | AY331593.1 | *C.* sp3 Oahu, Hawaii 3 |  |
|  | *Cassiopea* sp3 | *Cassiopea* sp3 | Kualoa Ranch, windward O’ahu | AY331595.1 | *C.* sp3 Oahu, Hawaii 4 |  |
|  | *C. andromeda* | *C. andromeda* | Hilton lagoon, Waikiki, leeward O’ahu | AF231109.1 | *C. andromeda* Oahu, Hawaii |  |
|  | *C. andromeda* | *C. andromeda* | Kainaone fish pond, Moloka’i | AY319453.1 | *C. andromeda* Molokai, Hawaii 1 |  |
|  | *C. andromeda* | *C. andromeda* | Kainaone fish pond, Moloka’i | AY319454.1 | *C. andromeda* Molokai, Hawaii 2 |  |
|  | *C. ornata* | *C. ornata* | Short Drop Off, Palau | AY319455.1 | *C. ornata* Palau 1 |  |
|  | *C. ornata* | *C. ornata* | Short Drop Off, Palau | AY319456.1 | *C. ornata* Palau 2 |  |
|  | *C. ornata* | *C. ornata* | Dravuni, Fiji | AY319457.1 | *C. ornata* Fiji |  |
|  | *Cassiopea* sp2 | *Cassiopea* sp2 | Observation Point, Papua New Guinea | AY319459.2 | *C.* sp2 Observation Point, PNG 1 |  |
|  | *Cassiopea* sp2 | *Cassiopea* sp2 | Observation Point, Papua New Guinea | AY319460.1 | *C.* sp2 Observation Point, PNG 2 |  |
|  | *Cassiopea* sp3 | *Cassiopea* sp3 | Emona, Papua New Guinea | AY319461.1 | *C.* sp3 Emona, PNG 1 |  |
|  | *Cassiopea* sp3 | *Cassiopea* sp3 | Emona, Papua New Guinea | AY319462.1 | *C.* sp 3 Emona, PNG 2 |  |
|  | *C. andromeda* | *C. andromeda* | El Ghardaqa, Egypt, Red Sea | AY319458.1 | *C. andromeda* Egypt |  |
|  | *Cassiopea* sp1 | *Cassiopea* sp1 | Port Douglas, Queensland, Australia | AY319471.1 | *C.* sp1 Port Douglas, Australia |  |
|  | *C. ornata* | *C. ornata* | Kakaban, Kalimantan, Indonesia | AY319472.1 | *C. ornata* Indonesia |  |
|  | *C. frondosa* | *C. frondosa* | San Blas Islands, Panama | AY319469.1 | *C. frondosa* Panama 1 |  |
|  | *C. frondosa* | *C. frondosa* | San Blas Islands, Panama | AY319470.1 | *C. frondosa* Panama 2 |  |
|  | *C. frondosa* | *C. frondosa* | Key Largo, Florida Keys | AY319467.1 | *C. frondosa* Florida Keys |  |
|  | *C. andromeda* | *C. andromeda* | Key Largo, Florida Keys | AY319468.1 | *C. andromeda* Florida Keys |  |
|  | *C. andromeda* | *C. andromeda* | Walsingham Pond, Bermuda | AY319463.1 | *C. andromeda* Walsingham Pond, Bermuda 1 |  |
|  | *C. andromeda* | *C. andromeda* | Walsingham Pond, Bermuda | AY319466.1 | *C. andromeda* Walsingham Pond, Bermuda 2 |  |
|  | *C. andromeda* | *C. andromeda* | Richardson Bay, Bermuda | AY319464.1 | *C. andromeda* Richardson Bay, Bermuda 1 |  |
|  | *C. andromeda* | *C. andromeda* | Richardson Bay, Bermuda | AY319465.1 | *C. andromeda* Richardson Bay, Bermuda 2 |  |
|  | *C. ornata* | *C. ornata* | South Cassiopea Lake, Palau | LC198724.1 | *C. ornata* Palau 3 | Arai et al (2017) Phylogenetic relationships and morphological variations of upsidedown jellyfishes, Cassiopea spp. inhabiting Palau Island |
|  | *C. ornata* | *C. ornata* | NGE Lake 2, Palau | LC198725.1 | *C. ornata* Palau 4 |  |
|  | *C. ornata* | *C. ornata* | NGE Lake 1 Lagoon, Palau | LC198726.1 | *C. ornata* Palau 5 |  |
|  | *C. ornata* | *C. ornata* | NGE Lake 1 Lagoon, Palau | LC198727.1 | *C. ornata* Palau 6 |  |
|  | *C. ornata* | *C. ornata* | Milky Way Lake 2, Palau | LC198728.1 | *C. ornata* Palau 7 |  |
|  | *C. ornata* | *C. ornata* | Milky Way Lake 2, Palau | LC198729.1 | *C. ornata* Palau 8 |  |
|  | *C. ornata* | *C. ornata* | Milky Way Lake 2, Palau | LC198730.1 | *C. ornata* Palau 9 |  |
|  | *C. ornata* | *C. ornata* | Milky Way Lake 2, Palau | LC198731.1 | *C. ornata* Palau 10 |  |
|  | *C. ornata* | *C. ornata* | Milky Way Lake 2, Palau | LC198732.1 | *C. ornata* Palau 11 |  |
|  | *C. andromeda* | *C. andromeda* | T Lake, Palau | LC198774.1 | *C. andromeda* Palau |  |
|  | *Cassiopea* sp4 | *Cassiopea* sp4 | Milky Way Lake 1, Palau | LC198739.1 | *C.* sp4 Palau 1 |  |
|  | *Cassiopea* sp4 | *Cassiopea* sp4 | Ongael Lake, Palau | LC198740.1 | *C.* sp4 Palau 2 |  |
|  | *Cassiopea* sp4 | *Cassiopea* sp4 | Ongael Lake, Palau | LC198741.1 | *C.* sp4 Palau 3 |  |
|  | *Cassiopea* sp4 | *Cassiopea* sp4 | Ongael Lake, Palau | LC198742.1 | *C.* sp4 Palau 4 |  |
|  | *Cassiopea* sp4 | *Cassiopea* sp4 | Ongael Lake, Palau | LC198743.1 | *C.* sp4 Palau 5 |  |
|  | *Cassiopea* sp4 | *Cassiopea* sp4 | Ongael Lake, Palau | LC198744.1 | *C.* sp4 Palau 6 |  |
|  | *Cassiopea* sp4 | *Cassiopea* sp4 | T Lake, Palau | LC198745.1 | *C.* sp4 Palau 7 |  |
|  | *Cassiopea* sp4 | *Cassiopea* sp4 | T Lake, Palau | LC198746.1 | *C.* sp4 Palau 8 |  |
|  | *Cassiopea* sp4 | *Cassiopea* sp4 | T Lake, Palau | LC198747.1 | *C.* sp4 Palau 9 |  |
|  | *Cassiopea* sp4 | *Cassiopea* sp4 | T Lake, Palau | LC198748.1 | *C.* sp4 Palau 10 |  |
|  | *Cassiopea* sp4 | *Cassiopea* sp4 | T Lake, Palau | LC198749.1 | *C.* sp4 Palau 11 |  |
|  | *Cassiopea* sp4 | *Cassiopea* sp4 | T Lake, Palau | LC198750.1 | *C.* sp4 Palau 12 |  |
|  | *Cassiopea* sp4 | *Cassiopea* sp4 | T Lake, Palau | LC198751.1 | *C.* sp4 Palau 13 |  |
|  | *Cassiopea* sp4 | *Cassiopea* sp4 | T Lake, Palau | LC198752.1 | *C.* sp4 Palau 14 |  |
|  | *Cassiopea* sp4 | *Cassiopea* sp4 | NGE Lake 1 Lagoon, Palau | LC198753.1 | *C.* sp4 Palau 15 |  |
|  | *Cassiopea* sp6 | *Cassiopea* sp6 | NGE Lake 1 Lagoon, Palau | LC198754.1 | *C.* sp6 Palau |  |
|  | *C. andromeda* | *C. andromeda* | Araruama Langoon, Brazil | KC464458.1 | *C. andromeda* Brazil 1 | Morandi et al (2017) All non-indigenous species were introduced recently? The case study of Cassiopea (Cnidaria: Scyphozoa) in Brazilian waters |
|  | *C. andromeda* | *C. andromeda* | Isla San Jose, Baja California Sur, Mexico | KY610551.1 | *C. andromeda* Mexico | Daglio & Dawson (2017) Species richness of jellyfishes (Scyphozoa: Discomedusae) in the Tropical Eastern Pacific: missed taxa, molecules, and morphology match in a biodiversity hotspot |
|  | *C. xamachana* | *C. frondosa* | Bahia Delfines, Bocas del Toro, Panama | KY610560.1 | *C. xamachana* Panama 1 |  |
|  | *C. frondosa* | *C. xamachana* | Bahia Delfines, Bocas del Toro, Panama | KY610559.1 | *C. frondosa* Panama 3 |  |
|  | *C. frondosa* | *C. xamachana* | Pacific Ocean, Northwest Pacific | HF930519.1 | *C. frondosa* Northwest Pacific Ocean | Armani et al (2013) What is inside the jar? Forensically informative nucleotide sequencing (FINS) of a short mitochondrial COI gene fragment revelas a high percentage of mislabeling in jellyfish food products |
|  | *C. andromeda* | *C. andromeda* | Western Indian Ocean | HF930521.1 | *C. andromeda* Western Indian Ocean |  |
|  | *C. andromeda* | *C. andromeda* | Pratagy, Maceió, Alagoas, Brazil | MT806178.1 | *C. andromeda* Brazil 2 | Stampar et al (2021) The puzzling occurrence of the upside-down jellyfish Cassiopea (Cnidaria: Scyphozoa) along the Brazilian coast: a result of several invasion events? |
|  | *C. andromeda* | *C. andromeda* | Pratagy, Maceió, Alagoas, Brazil | MT806179.1 | *C. andromeda* Brazil 3 |  |
|  | *C. andromeda* | *C. andromeda* | Pratagy, Maceió, Alagoas, Brazil | MT806180.1 | *C. andromeda* Brazil 4 |  |
|  | *C. andromeda* | *C. andromeda* | Pratagy, Maceió, Alagoas, Brazil | MT806181.1 | *C. andromeda* Brazil 5 |  |
|  | *C. xamachana* | *C. xamachana* | São Sebastião, São Paulo, Brazil | MN539722.1 | *C. xamachana* Brazil 1 |  |
|  | *C. xamachana* | *C. xamachana* | Imbé, Rio Grande do Sul, Brazil | MN602311.1 | *C. xamachana* Brazil 2 | Gamero-Mora (2019) Regenerative capacity of the upside-down jellyfish Cassiopea xamachana |
|  | *C. xamachana* | *C. xamachana* | unkown | MZ343250.1 | *C. xamachana* unkown location | Muffett & Miglietta (unpublished) Development of Cassiopea in low PAR conditions |
|  | *C. xamachana* | *C. xamachana* | Coconut Island, Kaneohoe Bay, Oahu, Hawaii | MW277725.1 | *C. xamachana* Oahu, Hawaii 1 | Pauley et al (unpublished) Smithsonian Institute DNA barcoding Marine Invertebrates |
|  | *C. xamachana* | *C. xamachana* | Coconut Island, Kaneohoe Bay, Oahu, Hawaii | MW277722.1 | *C. xamachana* Oahu, Hawaii 2 |  |
|  | *C. xamachana* | *C. frondosa* | Bahia Delfines, Bocas del Toro, Panama | KY610562.1 | *C. xamachana* Panama 2 | Daglio & Dawson (2017) Species richness of jellyfishes (Scyphozoa: Discomedusae) in the Tropical Eastern Pacific: missed taxa, molecules, and morphology match in a biodiversity hotspot |
|  | *C. xamachana* | *C. frondosa* | Bahia Delfines, Bocas del Toro, Panama | KY610561.1 | *C. xamachana* Panama 3 |  |
|  | *Cassiopea* sp3 | *Cassiopea* sp3 | Lake Alexander, Northern Territory, Australia | MF742136.1 | *C.* sp3 Lake Alexander, Australia 1 | Abboud et al (2018) A global estimate of genetic and geographic differentiation in macromedusae implications for identifying the cause of jellyfish blooms |
|  | *Cassiopea* sp3 | *Cassiopea* sp3 | Lake Alexander, Northern Territory, Australia | MF742137.1 | *C.* sp3 Lake Alexander, Australia 2 |  |
|  | *Cassiopea* sp3 | *Cassiopea* sp3 | Lake Alexander, Northern Territory, Australia | MF742138.1 | *C.* sp3 Lake Alexander, Australia 3 |  |
|  | *Cassiopea* sp3 | *Cassiopea* sp3 | Coombabah Creek, Queensland, Australia | MF742133.1 | *C.* sp3 Coombabah Creek, Australia 2 |  |
|  | *Cassiopea* sp3 | *Cassiopea* sp3 | Coombabah Creek, Queensland, Australia | MF742134.1 | *C.* sp3 Coombabah Creek, Australia 3 |  |
|  | *Cassiopea* sp3 | *Cassiopea* sp3 | Coombabah Creek, Queensland, Australia | MF742135.1 | *C.* sp3 Coombabah Creek, Australia 4 |  |
|  | *Cassiopea* sp3 | *Cassiopea* sp3 | Haji Buang, Maratua, Indonesia | MF742143.1 | *C.* sp Indonesia |  |
|  | *Cassiopea* sp3 | *Cassiopea* sp3 | Kagoshima Bay, Japan | MF742162.1 | *C.* sp3 Japan 1 |  |
|  | *Cassiopea* sp3 | *Cassiopea* sp3 | Kagoshima Bay, Japan | MF742163.1 | *C.* sp3 Japan 2 |  |
|  | *Cassiopea* sp3 | *Cassiopea* sp3 | Kagoshima Bay, Japan | MF742164.1 | *C.* sp3 Japan 3 |  |
|  | *Cassiopea* sp | *Cassiopea* sp | Mo'orea, Windward Islands, French Polynesia | MF742213.1 | *C.* sp French Polynesia |  |
|  | *Cassiopea* sp | *Cassiopea* sp | Nggatokae Mangroves, Western Solomon Island | MF742187.1 | *C.* sp Solomon Islands |  |
|  | *Cassiopea* sp | *Cassiopea* sp | Key Largo, Florida Keys | MF742149.1 | *C.* sp Florida Keys |  |
|  | *Cassiopea* sp | *Cassiopea* sp | Kri, Sorido Bay, Papua | MF742207.1 | *C.* sp Papua |  |
|  | *C. ornata* | *C. culionesnsis* | Monterey Bay Aquarium, California, USA | KF683387.1 | *C. ornata* California | Mellas et al. (2014) Symbiosis flexibility and effects of Symbiodinium identifity in the upside-down jellyfish Cassiopea sp. |
|  | *C. andromeda* | *C. mayeri* | Enoshima Aquarium, Japan? | AB563740.1 | *C. andromeda* Japan | Ojimi and Hidaka (2010) Comparison of telomere length among different life cycle stages of the jellyfish Cassiopea andromeda |
|  | *Catostylus mosaicus* | *Catostylus mosaicus* | Port Albert, Victoria, Australia | AY737222.1 | *Catostylus mosaicus* | Dawson (2005) Independent speciation of Catostylus mosaicus (Scyphozoa: Rhizostomeae: Catostlyidae), comparative phylogeography, and biogeography in southeast Australia |
|  | *Aurelia aurita* | *Aurelia aurita* | Boston Harbour,Massachusetts, USA | AY903093.1 | *Aurelia aurita* | Dawson (2005) Coupled biophysical global ocean model and molecular genetic analyses identify multiple introductions of cryptogenic species |
