# Supplementary material for: Identification of Cassiopea sp. in Lake Macquarie, Australia and revision of the taxonomic status of Cassiopea maremetens Gershwin, Zeidler & Davie, 2010 (Cnidaria: Scyphozoa: Cassiopeidae)
Source: PeerJ. 2025 Jul 18;13:e19669. doi: 10.7717/peerj.19669 (PMC12278942; doi:10.7717/peerj.19669)
Supplement: Supplemental Information 3 [file peerj-13-19669-s003.docx]

| **AM rego #** | **Location** | **Species** | **Institution** | **# of specimens** | **Locality** | **GPS Location** | **Date Collected** | **Type material** | **Preservative** | **Date examined** |
| --- | --- | --- | --- | --- | --- | --- | --- | --- | --- | --- |
| G.20057 | Coombabah Creek, QLD | C. sp | Australian Museum | 1 | Australia, Queensland, Gold Coast, Coombabah Creek, Kangaroo Ave | 27° 54' 26" S, 153° 22' 57" E | 8-Mar-21 |  | Formalin | 1/04/2021 |
| G.20058 | Coombabah Creek, QLD | C. sp | Australian Museum | 1 | Australia, Queensland, Gold Coast, Coombabah Creek, Kangaroo Ave | 27° 54' 26" S, 153° 22' 57" E | 8-Mar-21 |  | Formalin | 1/04/2021 |
| G.20059 | Coombabah Creek, QLD | C. sp | Australian Museum | 1 | Australia, Queensland, Gold Coast, Coombabah Creek, Kangaroo Ave | 27° 54' 26" S, 153° 22' 57" E | 8-Mar-21 |  | Formalin | 30/03/2021 |
| G.20060 | Coombabah Creek, QLD | C. sp | Australian Museum | 1 | Australia, Queensland, Gold Coast, Coombabah Creek, Kangaroo Ave | 27° 54' 27" S, 153° 22' 58" E | 9-Mar-21 |  | Formalin | 1/04/2021 |
| G.20061 | Coombabah Creek, QLD | C. sp | Australian Museum | 1 | Australia, Queensland, Gold Coast, Coombabah Creek, Kangaroo Ave | 27° 54' 27" S, 153° 22' 58" E | 9-Mar-21 |  | Formalin | 30/03/2021 |
| G.17363 | Lake Alexander, NT | C. sp | Australian Museum | 2 | Australia, Northern Territory, Darwin, Lake Alexander | 12° 24' S, 130° 49' E | 15-Nov-03 |  | Formalin | 22/04/2021 |
| G.17374 | Lake Alexander, NT | C. sp | Australian Museum | 1 | Australia, Northern Territory, Darwin, Lake Alexander | 12° 24' S, 130° 49' E | 15-Nov-03 |  | Formalin | 22/04/2021 |
| G.18075 | Lake Illawarra, NSW | *C. sp* | Australian Museum | 1 | Australia, New South Wales, Lake Illawarra, canal through Jetties By The Lake at Windang Road | 34° 31' 36" S, 150° 51' 53" E | 08/05/2013 |  | Formalin | 03/03/2021 |
| G.20077 | Lake Illawarra, NSW | C. sp | Australian Museum | 11 | Australia, New South Wales, Lake Illawarra, canal through Jetties by the lake at Windang Road | 34° 31' 36" S, 150° 51' 52" E | 1-Jun-21 |  | Formalin | 24/09/2021 |
| G.20076 | Port Hacking, NSW | *C. sp* | Australian Museum | 10 | Australia, New South Wales, channel between rocky shore and sand spit, north east of entrance to Cabbage Tree Basin, near entrance to Port Hacking | 34° 04' 45" S, 151° 07' 59" E | 31/05/2021 |  | Formalin | 26/03/2023 |
| G.17370 | Papua New Guinea | C. sp2 | Australian Museum | 1 | Papua New Guinea, west side of Mascot Channel mouth, outer slope | 2° 40' 4" S, 150° 25' 58" E | 3-Jul-03 |  | Formalin | 22/04/2021 |
| G.17385 | Papua New Guinea | C. sp2 | Australian Museum | 5 | Papua New Guinea, west side of Mascot Channel mouth, outer slope | 2° 40' 4" S, 150° 25' 58" E | 3-Jul-03 |  | Formalin | 22/04/2021 |
| G.17387 | Papua New Guinea | C. sp2 | Australian Museum | 1 | Papua New Guinea, Manus, Sherburne Reef, atoll approx. 70 nautical miles south-south-east of Manus Island | 3° 19' 59" S, 148° 1' 3" E | 20-Jun-02 |  | Formalin | 22/04/2021 |
| G.18362 | Lake Macquarie, NSW | *C. xamachana* | Australian Museum | 9 | Australia, New South Wales, Lake Macquarie, Lake Petite | 33° 6' 59" S, 151° 32' 4" E | 4-Jun-17 |  | Formalin | 21/04/2021 |
| G.18363 | Lake Macquarie, NSW | *C. xamachana* | Australian Museum | 1 | Australia, New South Wales, Lake Macquarie, Lake Petite | 33° 06' 59" S, 151° 32' 04" E | 04/06/2017 |  | Formalin | 03/03/2021 |
| G.18364 | Lake Macquarie, NSW | *C. xamachana* | Australian Museum | 1 | Australia, New South Wales, Lake Macquarie, Lake Petite | 33° 06' 59" S, 151° 32' 04" E | 04/06/2017 |  | Formalin | 03/03/2021 |
| G.18365 | Lake Macquarie, NSW | *C. xamachana* | Australian Museum | 1 | Australia, New South Wales, Lake Macquarie, Lake Petite | 33° 6' 59" S, 151° 32' 4" E | 4-Jun-17 |  | Formalin | 13/04/2021 |
| G.18428 | Lake Macquarie, NSW | *C. xamachana* | Australian Museum | 1 | Australia, New South Wales, Lake Macquarie, Lake Petite | 33° 7' 0" S, 151° 31' 58" E | 9-Feb-18 |  | Formalin | 13/04/2021 |
| G.18528 | Lake Macquarie, NSW | *C. xamachana* | Australian Museum | 1 | Australia, New South Wales, Lake Macquarie, Karignan Creek | 33° 10' 36" S, 151° 34' 3" E | 16-May-18 |  | Formalin | 13/04/2021 |
| G.18720 | Lake Macquarie, NSW | *C. xamachana* | Australian Museum | 1 | Australia, New South Wales, Lake Macquarie, Karignan Creek | 33° 10' 32" S, 151° 34' 0" E | 6-May-21 |  | Formalin | 2/02/2021 |
| G.18721 | Lake Macquarie, NSW | *C. xamachana* | Australian Museum | 1 | Australia, New South Wales, Lake Macquarie, Karignan Creek | 33° 10' 32" S, 151° 34' 0" E | 6-May-21 |  | Formalin | 4/02/2021 |
| G.18722 | Lake Macquarie, NSW | *C. xamachana* | Australian Museum | 1 | Australia, New South Wales, Lake Macquarie, Karignan Creek | 33° 10' 32" S, 151° 34' 0" E | 6-May-19 |  | Formalin | 2/02/2021 |
| G.18723 | Lake Macquarie, NSW | *C. xamachana* | Australian Museum | 1 | Australia, New South Wales, Lake Macquarie, Karignan Creek | 33° 10' 32" S, 151° 34' 0" E | 6-May-21 |  | Formalin | 4/02/2021 |
| G.18724 | Lake Macquarie, NSW | *C. xamachana* | Australian Museum | 1 | Australia, New South Wales, Lake Macquarie, creek north of Kilaben Creek | 33° 1' 43" S, 151° 35' 1" E | 9-May-19 |  | Formalin | 2/02/2021 |
| G.18725 | Lake Macquarie, NSW | *C. xamachana* | Australian Museum | 1 | Australia, New South Wales, Lake Macquarie, creek north of Kilaben Creek | 33° 1' 43" S, 151° 35' 1" E | 9-May-21 |  | Formalin | 4/02/2021 |
| G.18726 | Lake Macquarie, NSW | *C. xamachana* | Australian Museum | 1 | Australia, New South Wales, Lake Macquarie, creek north of Kilaben Creek | 33° 1' 43" S, 151° 35' 1" E | 9-May-19 |  | Formalin | 27/01/2021 |
| G.18728 | Lake Macquarie, NSW | *C. xamachana* | Australian Museum | 1 | Australia, New South Wales, Lake Macquarie, creek north of Kilaben Creek | 33° 1' 43" S, 151° 35' 1" E | 9-May-19 |  | Formalin | 27/01/2021 |
| G.18730 | Lake Macquarie, NSW | *C. xamachana* | Australian Museum | 1 | Australia, New South Wales, Lake Macquarie, Lake Petite | 33° 7' 3" S, 151° 32' 6" E | 9-May-21 |  | Formalin | 2/02/2021 |
| G.18731 | Lake Macquarie, NSW | *C. xamachana* | Australian Museum | 1 | Australia, New South Wales, Lake Macquarie, Lake Petite | 33° 7' 3" S, 151° 32' 6" E | 9-May-19 |  | Formalin | 27/01/2021 |
| G.18732 | Lake Macquarie, NSW | *C. xamachana* | Australian Museum | 1 | Australia, New South Wales, Lake Macquarie, Lake Petite | 33° 7' 3" S, 151° 32' 6" E | 9-May-19 |  | Formalin | 27/01/2021 |
| G.18733 | Lake Macquarie, NSW | *C. xamachana* | Australian Museum | 1 | Australia, New South Wales, Lake Macquarie, Lake Petite | 33° 7' 3" S, 151° 32' 6" E | 9-May-19 |  | Formalin | 20/01/2021 |
| G.18734 | Lake Macquarie, NSW | *C. xamachana* | Australian Museum | 1 | Australia, New South Wales, Lake Macquarie, Lake Petite | 33° 7' 3" S, 151° 32' 6" E | 9-May-19 |  | Formalin | 20/01/2021 |
| G.18735 | Lake Macquarie, NSW | *C. xamachana* | Australian Museum | 1 | Australia, New South Wales, Lake Macquarie, Lake Petite | 33° 7' 3" S, 151° 32' 6" E | 9-May-19 |  | Formalin | 20/01/2021 |
| G.20068 | Pelican Waters, QLD | *C. xamachana* | Australian Museum | 1 | Australia, Queensland, Pelican Waters | 26° 50' 01" S, 153° 06' 44" E | 10-Mar-21 |  | Formalin | 30/03/2021 |
| G.20069 | Pelican Waters, QLD | *C. xamachana* | Australian Museum | 1 | Australia, Queensland, Pelican Waters | 26° 50' 01" S, 153° 06' 44" E | 10-Mar-21 |  | Formalin | 30/03/2021 |
| G.326486 | Pelican Waters. QLD | *C. xamachana* | Queensland Museum | 1 | Australia, Queensland, Pelican Waters, Lake Magellan, off Lamerough Canal | 26° 49' 47"S, 153° 6' 36" E | 24-May-07 | Holotype | Formalin | 12/02/2021 |
| G.18137 | Wallis Lake, NSW | *C. xamachana* | Australian Museum | 1 | Australia, New South Wales, Wallis Lake, Breckenridge Channel, in channel splitting Godwin Island approximately one third distance from southern shore | 32° 11' 45" S, 152° 29' 56" E | 15/08/2014 |  | Formalin | 04/03/2021 |
| G.18139 | Wallis Lake, NSW | *C. xamachana* | Australian Museum | 11 | Australia, New South Wales, Wallis Lake, Breckenridge Channel, in channel splitting Godwin Island approximately one third distance from southern shore | 32° 11' 45" S, 152° 29' 55" E | 15-Aug-14 |  | Formalin | 20/04/2021 |
| G.18144 | Wallis Lake, NSW | *C. xamachana* | Australian Museum | 1 | Australia, New South Wales, Wallis Lake, "The Keys", Pipers Creek, behind Smuggler's Cove Caravan Park | 32° 12' 0" S, 152° 30' 39" E | 18-Sep-14 |  | Formalin | 13/04/2021 |
| G.18148 | Wallis Lake, NSW | *C. xamachana* | Australian Museum | 1 | Australia, New South Wales, Wallis Lake, "The Keys", Pipers Creek, behind Smuggler's Cove Caravan Park | 32° 12' 00" S, 152° 30' 40" E | 18/09/2014 |  | Formalin | 04/03/2021 |
| G.18149 | Wallis Lake, NSW | *C. xamachana* | Australian Museum | 1 | Australia, New South Wales, Wallis Lake, "The Keys", Pipers Creek, behind Smuggler's Cove Caravan Park | 32° 12' 00" S, 152° 30' 40" E | 18/09/2014 |  | Formalin | 04/03/2021 |
| G.18152 | Wallis Lake, NSW | *C. xamachana* | Australian Museum | 1 | Australia, New South Wales, Wallis Lake, "The Keys", Pipers Creek, behind Smuggler's Cove Caravan Park | 32° 12' 0" S, 152° 30' 39" E | 18-Sep-14 |  | Formalin | 22/04/2021 |
| G.18153 | Wallis Lake, NSW | *C. xamachana* | Australian Museum | 1 | Australia, New South Wales, Wallis Lake, "The Keys", Pipers Creek, behind Smuggler's Cove Caravan Park | 32° 12' 00" S, 152° 30' 40" E | 18/09/2014 |  | Formalin | 04/03/2021 |
| G.18154 | Wallis Lake, NSW | *C. xamachana* | Australian Museum | 1 | Australia, New South Wales, Wallis Lake, "The Keys", Pipers Creek, behind Smuggler's Cove Caravan Park | 32° 12' 00" S, 152° 30' 40" E | 18/09/2014 |  | Formalin | 04/03/21 |
| G.18156 | Wallis Lake, NSW | *C. xamachana* | Australian Museum | 4 | Australia, New South Wales, Wallis Lake, "The Keys", Pipers Creek, behind Smuggler's Cove Caravan Park | 32° 12' 0" S, 152° 30' 39" E | 18-Sep-14 |  | Formalin | 22/04/2021 |
| G.18736 | Wallis Lake, NSW | *C. xamachana* | Australian Museum | 1 | Australia, New South Wales, Wallis Lake, Pipers Creek within Smugglers Cove Caravan Park | 32° 11' 58" S, 152° 30' 39" E | 10-May-19 |  | Formalin | 20/01/2021 |
| G.18738 | Wallis Lake, NSW | *C. xamachana* | Australian Museum | 1 | Australia, New South Wales, Wallis Lake, Pipers Creek within Smugglers Cove Caravan Park | 32° 11' 58" S, 152° 30' 39" E | 10-May-19 |  | Formalin | 20/01/2021 |
| G.18739 | Wallis Lake, NSW | *C. xamachana* | Australian Museum | 1 | Australia, New South Wales, Wallis Lake, Pipers Creek within Smugglers Cove Caravan Park | 32° 11' 58" S, 152° 30' 39" E | 10-May-19 |  | Formalin | 20/01/2021 |
| G.18741 | Wallis Lake, NSW | *C. xamachana* | Australian Museum | 1 | Australia, New South Wales, Wallis Lake, Pipers Creek within Smugglers Cove Caravan Park | 32° 11' 58" S, 152° 30' 39" E | 10-May-19 |  | Formalin | 19/01/2021 |
| G.18742 | Wallis Lake, NSW | *C. xamachana* | Australian Museum | 1 | Australia, New South Wales, Wallis Lake, Pipers Creek within Smugglers Cove Caravan Park | 32° 11' 58" S, 152° 30' 39" E | 10-May-19 |  | Formalin | 19/01/2021 |
| G.18745 | Wallis Lake, NSW | *C. xamachana* | Australian Museum | 1 | Australia, New South Wales, Wallis Lake, Pipers Creek within Smugglers Cove Caravan Park | 32° 11' 58" S, 152° 30' 39" E | 10-May-19 |  | Formalin | 19/01/2021 |
| G.18746 | Wallis Lake, NSW | *C. xamachana* | Australian Museum | 1 | Australia, New South Wales, Wallis Lake, Pipers Creek within Smugglers Cove Caravan Park | 32° 11' 58" S, 152° 30' 39" E | 10-May-19 |  | Formalin | 19/01/2021 |
| G.18748 | Wallis Lake, NSW | *C. xamachana* | Australian Museum | 1 | Australia, New South Wales, Wallis Lake, Pipers Creek within Smugglers Cove Caravan Park | 32° 11' 58" S, 152° 30' 39" E | 10-May-19 |  | Formalin | 19/01/2021 |
| G.18749 | Wallis Lake, NSW | *C. xamachana* | Australian Museum | 1 | Australia, New South Wales, Wallis Lake, Pipers Creek within Smugglers Cove Caravan Park | 32° 11' 58" S, 152° 30' 39" E | 10-May-19 |  | Formalin | 19/01/2021 |
| G.18750 | Wallis Lake, NSW | *C. xamachana* | Australian Museum | 1 | Australia, New South Wales, Wallis Lake, Pipers Creek within Smugglers Cove Caravan Park | 32° 11' 58" S, 152° 30' 39" E | 10-May-19 |  | Formalin | 22/09/2020 |
| G.18752 | Wallis Lake, NSW | *C. xamachana* | Australian Museum | 1 | Australia, New South Wales, Wallis Lake, Pipers Creek within Smugglers Cove Caravan Park | 32° 11' 58" S, 152° 30' 39" E | 10-May-19 |  | Formalin | 22/09/2020 |
| G.18753 | Wallis Lake, NSW | *C. xamachana* | Australian Museum | 1 | Australia, New South Wales, Wallis Lake, Pipers Creek within Smugglers Cove Caravan Park | 32° 11' 58" S, 152° 30' 39" E | 10-May-19 |  | Formalin | 22/09/2020 |
| G.18754 | Wallis Lake, NSW | *C. xamachana* | Australian Museum | 1 | Australia, New South Wales, Wallis Lake, Pipers Creek within Smugglers Cove Caravan Park | 32° 11' 58" S, 152° 30' 39" E | 10-May-19 |  | Formalin | 22/09/2020 |
| G.18755 | Wallis Lake, NSW | *C. xamachana* | Australian Museum | 1 | Australia, New South Wales, Wallis Lake, Pipers Creek within Smugglers Cove Caravan Park | 32° 11' 58" S, 152° 30' 39" E | 10-May-19 |  | Formalin | 22/09/2020 |
