# Supplementary figures and images for: Identification of Cassiopea sp. in Lake Macquarie, Australia and revision of the taxonomic status of Cassiopea maremetens Gershwin, Zeidler & Davie, 2010 (Cnidaria: Scyphozoa: Cassiopeidae)
Source: PeerJ. 2025 Jul 18;13:e19669. doi: 10.7717/peerj.19669 (PMC12278942; doi:10.7717/peerj.19669)

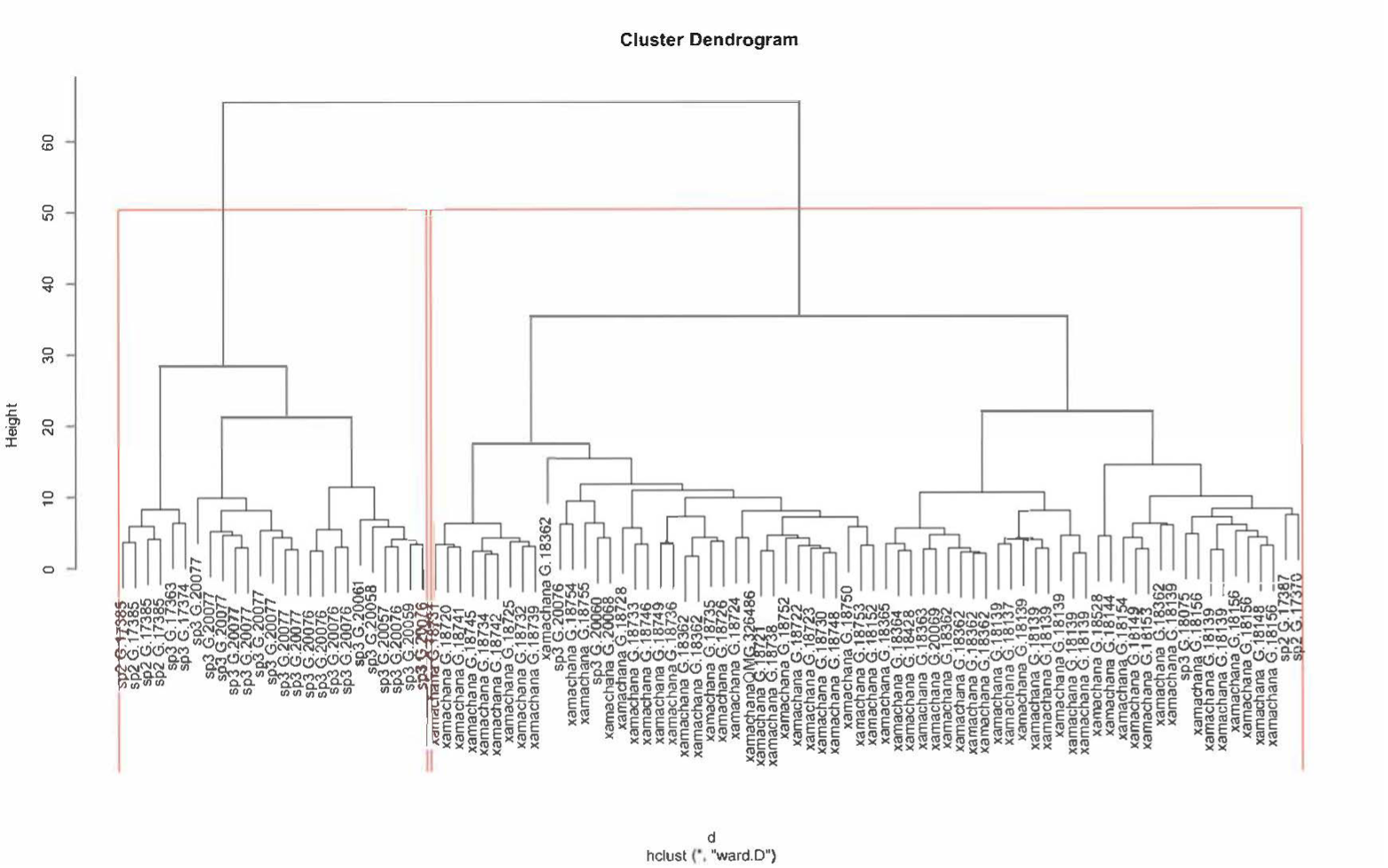

Supplement: Supplemental Information 6 — Red lines indicate morphologically homogeneous clusters detected by Ward Hierarchical Clustering and the k-means of the specimens. Labels indicate species and their registration number. All specimens from the AM, except for the Holotype (QM G.326486). [file peerj-13-19669-s006.png]

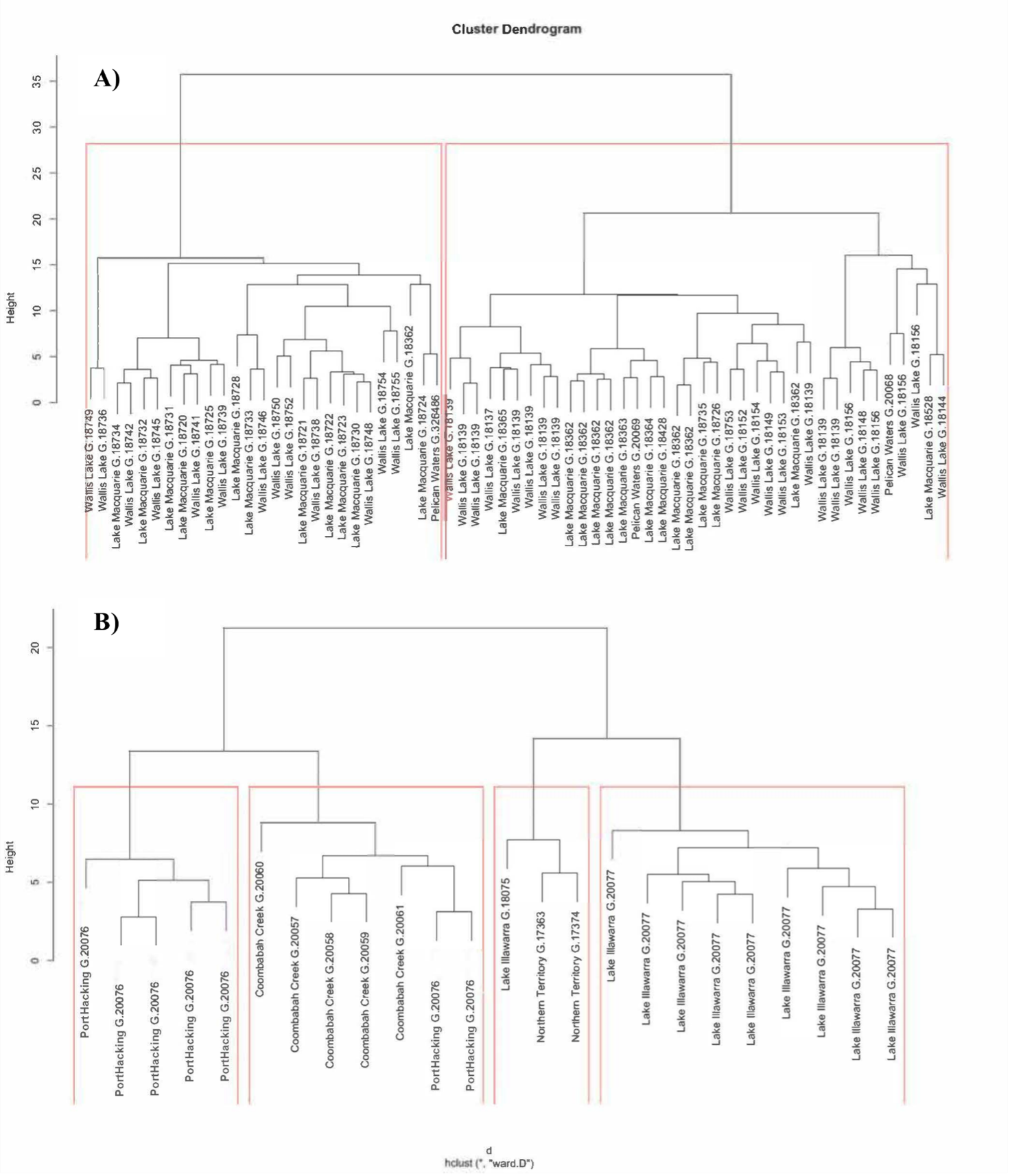

Supplement: Supplemental Information 7 — A) Cassiopea xamachana Bigelow, 1892. B) Cassiopea sp.3. Red lines indicate morphologically homogeneous clusters detected by Ward Hierarchical Clustering and the k- means of the specimens. Labels indicate the population and their AM registration number. [file peerj-13-19669-s007.png]
